# Maternal high-fat high-energy diet impairs surfactant maturation in the non-human primate fetal lung

**DOI:** 10.1038/s41366-026-02090-7

**Published:** 2026-04-27

**Authors:** Mitchell C. Lock, Hillary F. Huber, Cun Li, Sandra Orgeig, Peter W. Nathanielsz, Janna L. Morrison

**Affiliations:** 1 Early Origins of Adult Health Research Group, Adelaide, SA Australia; 2 Department of Animal Science, University of Wyoming, Laramie, WY USA; 3 Southwest National Primate Research Center, Texas Biomedical Research Institute, San Antonio, TX USA; 4 School of Pharmacy and Biomedical Sciences, College of Health, Adelaide University, Adelaide, SA Australia

**Keywords:** Physiology, Preclinical research

## Abstract

**Introduction:**

Due to the global obesity crisis, increasing numbers of women enter pregnancy with overweight or obesity. Their offspring are at greater risk of respiratory complications at birth due to metabolic changes that impact lung development that may reduce capacity for surfactant production. We hypothesize that a high-fat-high-energy diet (HF-HED) negatively impacts late gestation fetal lung development.

**Methods:**

Female baboons were randomly assigned to Control (metabolizable energy content, MEC = 3.07 kcal/g, 12% from fat; *n* = 5 M, 3 F fetuses) or high-fat high-energy diet (MEC = 4.03 kcal/g, 45% energy from fat; *n* = 6 M, 6 F fetuses) before and throughout pregnancy. Fetal lung tissue was collected at 0.9 gestation (term, 184 d). qRT-PCR and immunohistochemistry were utilized to measure expression of key molecules involved in surfactant maturation, the transition to air breathing.

**Results:**

HF-HED decreased fetal type-II alveolar epithelial cells and reduced lung surfactant protein expression (*SFTPB, SFTPC*, and *SFTPD*). The rate-limiting genes involved in surfactant phospholipid production *PCY1TA* and *ABCA3* was reduced. Genes involved in water (*AQP1*) and sodium (*ATP1A1* and *SCNN1B*) transport were also downregulated, indicating impaired lung liquid reabsorption.

**Conclusion:**

These data indicate that a maternal obesogenic diet impairs surfactant maturation and reduces the capacity for lung liquid reabsorption, increasing the risk of neonatal respiratory complications.

**Figure:**
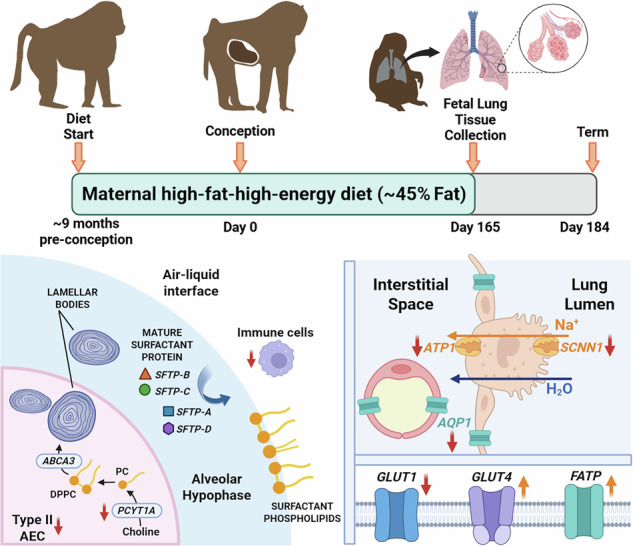
Summary of changes within the fetal lung due to exposure to a HF-HED throughout pregnancy. Surfactant maturation was negatively impacted, with a reduction in type II alveolar epithelial cell numbers, reduced mRNA expression in rate limiting enzymes in the production of surfactant phospholipids (*PCYT1A, ABCA3*), and reduced mRNA expression of surfactant proteins (*SFTPB, SFTPC, SFTPD*). There was also reduced mRNA expression of sodium transporters (*ATP1A1* and *SCNN1*) which would potentially negatively impact lung liquid reabsorption. Glucose and fatty acid metabolism were dysregulated in the fetal lung with downregulation of glucose transporter 1 (*GLUT1*) but upregulation of insulin-dependent-glucose and fatty acid transporters (*GLUT4, FATP1*) and fatty acid synthesis (*FASN*).

## Introduction

Respiratory distress syndrome (RDS) is one of the leading causes of death in the first month of life [1, 2]. In recent years it has become clear that there is an association between women who have obesity or overweight and having a greater risk of delivering a neonate with RDS [3–8]. During late gestation, pregnant women normally become insulin resistant in an effort to drive the transfer of glucose to the developing fetus. However, women consuming a Western-style diet that is high in fat and energy are more insulin resistant before pregnancy and remain so throughout pregnancy, leading to a higher risk of developing gestational diabetes mellitus (GDM), and therefore higher fetal glucose exposure. Clinical evidence has uncovered a close association between GDM and RDS at birth, irrespective of birth weight or preterm delivery [9–11]. We need a greater understanding of the relationship between maternal diet and fetal lung development to be able to successfully identify and treat at-risk individuals.

Pulmonary surfactant is a complex mixture of lipids and proteins that function to reduce surface tension at the air-liquid interface, preventing alveolar collapse and supporting innate immunity in the lung [12]. Sufficient pulmonary surfactant production is required at birth to allow for proper gas exchange, with impaired surfactant maturation being one of the main components in the pathogenesis of RDS [13]. Type II alveolar epithelial cells (AECII) are responsible for synthesis of both the lipid and protein components of pulmonary surfactant, which is stored in cytoplasmic inclusion bodies, known as lamellar bodies, and secreted into the alveolar fluid lining in response to a number of stimulatory factors to form the surface-active film [14]. Hence, the number of AECII’s within the alveolar epithelium is a major factor in the capacity for surfactant production by the neonate and is of critical importance to lung function. Previous evidence from our group and others has demonstrated both impaired surfactant production and reduced numerical density of AECII due to elevated glucose and insulin in fetal sheep and rats [15–17]. Another major factor involved in the pathogenesis of RDS is impaired lung liquid reabsorption at birth, often observed as transient tachypnea of the newborn (TTN) [18]. Prior to labor, lung fluid secretion is reduced and the onset of labor stimulates the production of adrenaline by the fetus and thyrotrophin-releasing hormone by the mother, causing fetal pulmonary epithelial cells to begin reabsorption of lung fluid [19]. After birth, there is an acceleration of active pulmonary fluid absorption, and most is cleared from the full-term newborn lung within 2 h of commencing spontaneous breathing. This is achieved by the active transport of sodium ions out of the alveolar lumen and into the interstitium [20]. To our knowledge the impact of maternal high-fat, high-energy diet (HF-HED) during pregnancy on the expression of water and sodium transporters in the lung has not been previously investigated in a primate or any other model.

The molecular mechanisms underlying the adverse effects of HF-HED during pregnancy are not well understood; the impact on the fetus involves a combination of factors related to the hyperglycemic and proinflammatory environment [21]. Hyperglycemia can induce oxidative stress and inflammation in the fetal lungs, further contributing to impaired lung development and an increased risk of RDS [22]. Furthermore, GDM can also lead to placental insufficiency, restricting oxygen and nutrient delivery to the fetus, further compromising lung development and increasing the susceptibility to RDS [23, 24]. We have previously investigated overnutrition in a sheep model during late pregnancy, which showed increased maternal and fetal plasma glucose and insulin concentrations [15]. But the impact of a high-fat Western-style diet on fetal lung development in a clinically relevant large animal model has not yet been well explored. Some evidence from rodent models of high-fat diet has demonstrated impaired fetal lung development likely to predispose offspring to increased risk of RDS at birth [25, 26]. However, rodent models of high-fat diet also result in substantial fetal growth restriction, dissimilar to the prevalence of macrosomia observed in humans [21]. Additionally, lung development in rodents is delayed relative to birth compared to large animals such as sheep, baboons, and humans [4]. Baboons are the closest translational model for human pregnancy in terms of their genetics, body size, placental structure and function, and reproductive physiology [27–30]. A deeper understanding of the intricate mechanisms involved in development of the fetal lung is crucial for the development of targeted interventions to mitigate the risk of RDS in infants born to mothers with obesity. We make use of baboon tissues from a biorepository assembled for a longitudinal study of the effects of maternal diet on offspring development [31–37]. We hypothesize that a maternal HF-HED in a clinically relevant non-human primate model will result in impaired maturation of the near-term fetal lung.

## Methods

### Ethics approval

All experimental protocols were approved by the Texas Biomedical Research Institute and University of Texas Health Science Center at San Antonio Institutional Animal Care and Use Committee, were conducted at the Southwest National Primate Research Center (AAALAC International accredited facility) and adhered to the United States Animal Welfare Act. All animal procedures were performed under the supervision of fully certified medical doctors or doctors of veterinary medicine (Protocol no. HD21350). The specific protocols for the acquisition, analysis, or interpretation of data within this manuscript were not pre-registered. The reporting of animal research in this manuscript adheres to the essential ARRIVE guidelines [38] and the principles of the 3Rs [39].

### Animal care, maintenance and nutritional diet

Baboons were maintained in groups of up to 16 in custom-built outdoor facilities allowing full socialization and free movement. Methods on the housing of animals, cage design, avoidance of confounding variables, exclusion criteria, and environmental enrichment have been reported [33, 36, 40]. Once stable groupings were confirmed, female baboons (*Papio hamadryas* spp.) of similar age (~11 years of age) and weight were randomly assigned to either a Control diet (*n* = 3 female (F), 5 male (M) offspring) or HF-HED (*n* = 6 F, 6 M offspring); fetal sex was unknown at the time of group assignment. Diets started ~9 months prior to mating and continued throughout pregnancy. The Control diet (Purina Monkey Diet 5LEO) consisted of 14% fat, 0.22% glucose, 0.24% fructose, and metabolizable energy content (MEC) of 2.98 kcal/g. *ad libitum*. The HF-HED consisted of 45% fat, 4.6% from glucose, 5.6% from fructose, and a MEC of 4.03 kcal/g *ad libitum* with free access to a high fructose beverage. Both diets were made available to the HF-HED group because baboons ate more HF-HED when it was provided together with the Control diet as previously described [36].

### Post-mortem and tissue collection

At 165 days gestation (term, ~184 d), Cesarean sections were performed under general anesthesia initiated with ketamine (7–10 mg/kg) and maintained with isoflurane (1–2% inhaled). The fetus was exteriorized from the uterus and humanely killed by exsanguination while under anesthesia, a method approved by the American Veterinary Medical Association. Detailed methods on Cesarean procedures and post-operative care and recovery period of female baboons are as previously described [41]. Fetal lung samples were collected and snap frozen in liquid nitrogen and stored at −80°C for quantitative real-time PCR or fixed in 4% paraformaldehyde solution for 48 h, dehydrated, and blocked in paraffin for immunohistochemical analysis.

### Real-time PCR for target genes

All essential information regarding the qRT-PCR procedure is included as per the MIQE guidelines [42]. Total RNA was extracted from frozen lung tissue for each fetus using QIAzol Lysis Reagent solution and QIAgen miRNeasy purification columns, as per manufacturer guidelines (Qiagen, Germany). Total RNA was quantified by spectrophotometric measurements at 260 and 280 nm in a NanoDrop Lite Spectrophotometer (Thermo Fisher Scientific). The 260/280 nm ratio results were less than 2.1 and greater than 1.9 and therefore acceptable for qRT-PCR. cDNA was synthesized using Superscript III First Strand Synthesis System (Invitrogen, USA) using 1 µg of total RNA, random hexamers, dNTP, DTT and Superscript III in a final volume of 20 µL, as per the manufacturer’s guidelines in a MiniAmp Plus Thermal Cycler (Thermo Fisher Scientific, USA). Controls containing either no RNA transcript or no Superscript III were used to test for reagent contamination and genomic DNA contamination, respectively. The geNorm component of qbaseplus 3.4 software (Biogazelle, Belgium) was used to determine the most stable reference genes from a panel of candidate reference genes [43]. and the minimum number of reference genes required to calculate a stable normalization factor, as previously described [44–46]. For qRT-PCR data output normalization, two stable reference genes TBP and ACTB (Table 1) were run in parallel with all target genes, as previously described [15]. A selection of genes was chosen a priori to investigate key pathways involved in lung development. Primers were validated and optimized as previously described [17, 23, 47]. Relative expression of target genes (Table 1) involved in: surfactant protein and phospholipid production (*SFTPA*, *SFTPB, SFTPC, SFTPD, PCYT1A, ABCA3, LPCAT1*), surfactant protein regulatory genes (*TTF1, SP1*), glucocorticoid signaling and conversion (*HSD11B1, HSD11B2*), water and sodium movement (*AQP1, AQP2, AQP4, ATP1A1, SCNN1B*), glucose transport (*GLUT1, GLUT4*), PPARγ signaling (*LEPR, PPARG, PPARGC1A*), fatty acid metabolism (*FASN, SLC27A1*), inflammation (*RELA, TNF*) and the immune response (*CD4, CD8, CD45, CD68*) were measured by qRT-PCR using KiCqStart SYBR Green qPCR ReadyMix (Sigma Aldrich, USA) in a final volume of 6 µL on a QuantStudio 7 Pro Real-time PCR system (Applied Biosystems, USA), as previously described [15]. Each qRT-PCR well contained 3 µL SYBR Green Master Mix (2X), 2 µL of forward and reverse primer mixed with H_2_O to obtain final primer concentrations and 1 µL of diluted cDNA. Each sample was run in triplicate for target and reference genes. The abundance of each transcript relative to the abundance of stable reference genes [48]. was calculated and expressed as mRNA mean normalized expression (MNE) ± SD.

**Table 1:** qRT-PCR primer list.

Primer name	Gene symbol	Reference/primer sequence 5′ to 3′	Accession number
Reference genes			
TATA-box binding protein	TBP	Fwd: GCTTCAGAGAGTTCTGGGATTGRev: GTGGTTCGTGGCTCTCTTATC	XM_009206461.4
Actin beta	ACTB	Fwd: GTCCACCGCAAATGCTTCTARev: CACCTTCACCGTTCCAGTTT	XM_003895688.4
Surfactant maturation			
Surfactant protein A1	SFTPA1	Fwd: CCTCCAGATGTGACCAGATTAG Rev: CTAAGACCTGGCACACGATAA	XM_003903711.5
Surfactant protein B	SFTPB	Fwd: GGATCTCTCTGAGCAGCAATTC Rev: TCGAGCAGGATGACAGAGTAG	XM_009184574.4
Surfactant protein C	SFTPC	Fwd: GTCTCCACATGAGCCAGAAA Rev: GCCGCTGGTAGTCATACAC	XM_003902511.3
Surfactant protein D	SFTPD	Fwd: GAGCATGACTGACTCCAAGATG Rev: CAAGCCCTGTCATTCCACTT	XM_003903708.4
Phosphate cytidylyltransferase 1, choline, alpha	PCYT1A	Fwd: CAACACAGAGGACAGAAGGTATC Rev: CCTCTCCTGCAAGTGGTATTT	XM_009202040.4
ATP-binding cassette sub-family A member 3	ABCA3	Fwd: CTGCTGGACTCTGTGCTTTAT Rev: CTTTCTCAGGGTCACTGTCTTC	XM_003916379.4
Lysophosphatidylcholine acyltransferase 1	LPCAT1	Fwd: CCTCATGACACTGACACTCTTC Rev: CTTCAGCAGGAAGTCCACAA	XM_003899463.4
Surfactant protein regulatory genes			
Sp1 transcription factor	SP1	Fwd: CCAATGAGAACAGCAACAACTC Rev: CACTCTGTTCCTTTGAGGTAGG	XM_031650504.1
Transcription termination factor 1	TTF1	Fwd: CTTTGGGAGTGAGAAGATGGAG Rev: TCAGAAACTGGAGTGTGGATTT	XM_031654672.1
Glucocorticoid signaling and conversion			
Hydroxysteroid 11-beta dehydrogenase 1	11BHSD1	Fwd: GGCTGGGAAAGTGGCTTAT Rev: ATGGCTGTGTCTGTGTCTATG	XM_009188051.3
Hydroxysteroid 11-beta dehydrogenase 2	11BHSD2	Fwd: TGACCAAACCAGGAGACATTAG Rev: TCCATGCAGCTACGGAAAG	XM_003917042.3
Water and sodium movement			
Aquaporin 1	AQP1	Fwd: CGTGGAGGAGGTGAAAGAAA Rev: CCATGACCAAAGGCACAAAG	XM_009203156.3
Aquaporin 2	AQP2	Fwd: CCAGCCTGCTGTGTAAGTAATA Rev: TGGAAGCAGAAAGGAGAAAGAG	XM_009180698.4
Aquaporin 3	AQP3	Fwd: ACCGAGCAAGAGAATGTGAAG Rev: CCTTAGCCTGGAAGGGTAGAT	XM_003911599.4
Aquaporin 4	AQP4	Fwd: GGTGTGCACCAGGAAGATAA Rev: CCATGACCAGCGGTAAGATT	XM_003914257.4
ATPase Na+/K+ transporting subunit alpha 1	ATP1A1	Fwd: GTGTTCCTGGGTGTGTCTTT Rev: ATGCGTTTGGCGGTAAGT	XM_021922594.2
Sodium channel epithelial 1 beta subunit	SCNN1B	Fwd: TGGTGTGGTACTGCGATAAC Rev: GCTCAAGTAGGTCCTGATGAAG	XM_009196153.4
Glucose transport			
Glucose transporter 1	GLUT1	Fwd: TCCTCATCGCCCAGGTATT Rev: CAGCTTCTTCAGCACACTCTT	NM_001173980.1
Glucose transporter 4	GLUT4	Fwd: CCTCAGAAGGTGATTGAACAGA Rev: CCAAGCCACTGAGAGATGATAC	XM_003912229.4
Fatty acid synthesis and transport			
Fatty acid synthase	FASN	Fwd: CAACCTGATAGTGAGTGGGAAG Rev: CATGGAAGTGAGGGCCATAG	XM_031657185.1
Solute carrier family 27 member 1	SLC27A1 (FATP1)	Fwd: GATCAAGTTCTGCTCTGGAGAC Rev: TGGTCCCTGACGTGTAGAT	XM_009193831.4
PPARγ signaling pathway			
Leptin receptor	LEPR	Fwd: CCAACAACTGTGGTCTCTCTAC Rev: CTGATCAGCGTGGCATATTTAAC	XM_031669581.1
Peroxisome proliferator activated receptor gamma	PPARG	Fwd: GCACCCTAGGCAAGATCTAAAG Rev: GCATGTGACTATCCCTGTCTTC	XM_021934188.2
PPARG coactivator 1 alpha	PPARGC1A	Fwd: TTGACGACGAAGCAGACAAG Rev: CCCAAGGGTAGCTCAGTTTATC	XM_031664083.1
Immune markers			
NF-kB subunit (RELA)	RELA	Fwd: CGAGCTTGTAGGAAAGGACTG Rev: AGGGATTGTTGTTCGTCTGG	XM_021925545.2
Tumor necrosis factor	TNF	Fwd: CTCTTCTGTCTGTTGCACTTTG Rev: TCAGCTTGAGGGTTTGCTAC	NM_001112648.1
Cluster of differentiation 4	CD4	Fwd: GCAGTTACCCAGGGAAAGAAA Rev: CTCAGCTTGGATGGACCTTTAG	XM_003905871.4
Cluster of differentiation 7	CD7	Fwd: GGCCTGCATGGGATCTAC Rev: CAGGTGGTGCATGGTAATAGT	XM_003913611.5
Protein tyrosine phosphatase receptor type C (PTPRC)	CD45	Fwd: GAGACCACACCATCTCTTAGTTC Rev: CTGCGTGTCAGTTCCAGTAG	XM_021928947.2
Cluster of differentiation 68	CD68	Fwd: GAGACCACACCATCTCTTAGTTC Rev: CTGCGTGTCAGTTCCAGTAG	XM_021928947.2

### Quantification of Pro-SFTP-B, Cleaved-Caspase 3, CD45+ positive cells and αSMA positive vessels within the fetal lung

Immunohistochemistry was performed using a rabbit anti-human mature pro-SFTP-B (1:300, WRAB-55522, Seven Hills Bioreagents, USA) Cleaved-Caspase 3 (1:400, ASP175, Cell Signaling Technology, Danvers, MA, USA), CD45 (1:500, ab10558, Abcam, Cambridge, UK) or anti-αSMA antibody (1:3000, MA106110, Thermo Fisher Scientific, USA) as previously described [49–51]. All sections were counterstained with Mayer’s Hematoxylin (Sigma Aldrich, USA). Negative Control slides (primary antibody negative control, and rabbit serum negative control) were incubated overnight at 4 °C in parallel with test slides under the same experimental conditions as described previously [49–51]. Stained sections (Control *n* = 8, HF-HED *n* = 12) were examined using Visiopharm new Computer Assisted Stereological Toolbox (NewCAST) software (Visiopharm, Denmark) as described previously [49–51]. Sixty counting frames (40× magnification) of the alveolar epithelium were randomly selected per tissue section. Positive staining was confirmed with the presence of cuboidal shaped cells exhibiting cytoplasmic staining within the alveolar epithelium of lung tissue sections. Point-counting using an unbiased counting frame with an area of 20,000 μm^2^ (pro-SPB) or 40,000 μm^2^ (αSMA) on immersion fixed tissue was used to estimate the numerical density of pro-SFTP-B positive cells and αSMA positive vessels within the alveolar epithelium of fetal lung tissue sections as described previously [49–52]. This method of analysis takes into account the number of points of the counting frame falling on lung tissue in each field of view and therefore inherently corrects for any differences in lung tissue density.

$$proSp{-}B\,positive\,cells\,per\,m{m}^{{2}}\,of\,lung\,tissue{=}\frac{{\sum }{Q}^{{-}}{(}Surfactant\,positive{)}}{{\sum }P\,{(}Lung\,tissue{)}\times \left[\frac{a{(}{f}{r}{a}{m}{e}{)}}{P}\right]}\times {{10}}^{{6}}$$

where Σ*Q*^−^ (Surfactant positive) represents the total number of SP-positive cells counted in all counting frames of one fetal lung tissue section, and Σ*P* (Lung tissue) represents the total number of points falling on lung tissue in each field of view. *P* is the number of points that were used to count the points included within the reference space (four corners per counting frame), and *a* is the total area of the counting frame. Cleaved caspase 3 and CD45+ staining were quantified using QuPath (v0.5.1) positive cell detection tool with the region of interest covering the entire tissue section. Correct detection of positive cells was confirmed on each slide by an experienced user blinded to the treatment groups.

### Statistical analysis

All data are presented as mean ± standard deviation (SD). Normality of the data was assessed using the Shapiro-Wilk test for each measure. The effects of treatment (Control, HF-HED) and sex were examined using a two-way analysis of variance (GraphPad Prism version 10, USA). As there was no significant effect of sex on the examined parameters and there were only 3 females in the control group, the data were reanalyzed using an unpaired Students’ *t* test between the Control and HF-HED groups (GraphPad Prism version 10, USA) and the *P*-values were adjusted for multiple comparisons using Bonferroni-Dunn correction for Type-I errors. Variance was not significant between the treatment groups (F-test, *P* > 0.05). Outliers were identified using the ROUT method with a *Q* = 1%. A *P*-value of <0.05 was considered significant.

## Results

### Fetal morphometric measurements

**Fig. 1:**
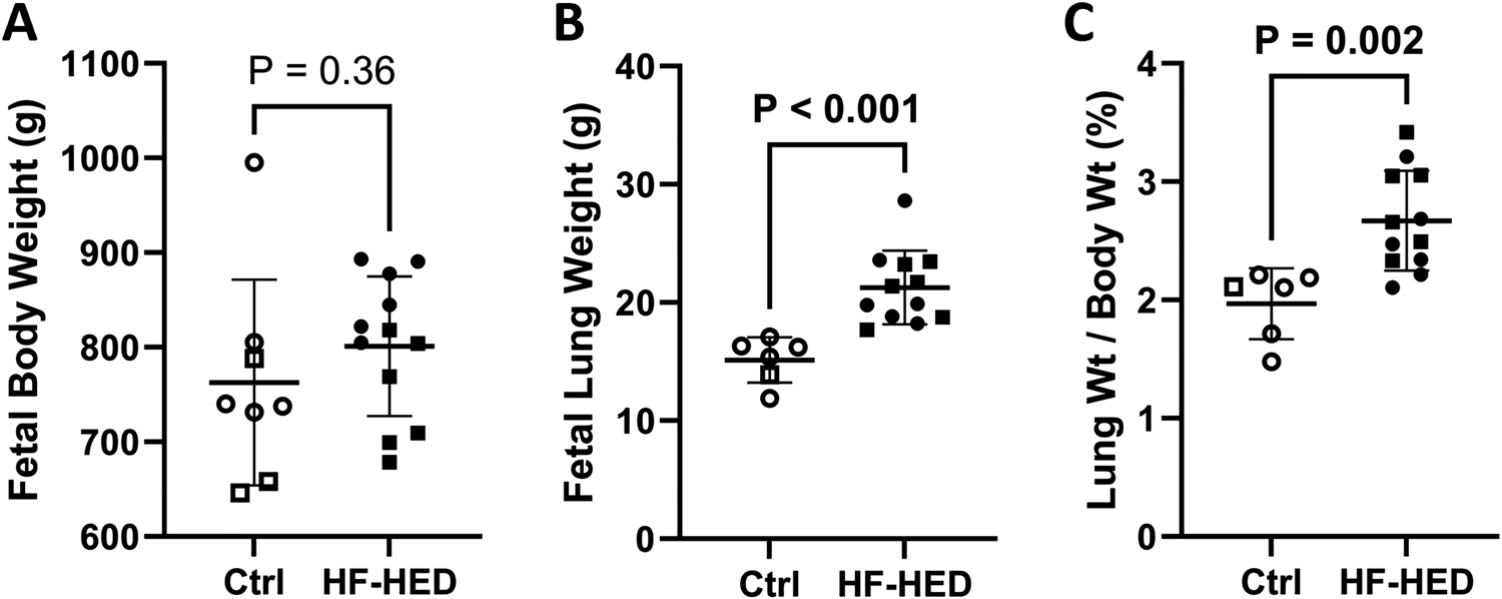
Fetal body parameters. **A** fetal body weight, **B** fetal lung weight and **C** lung weight to body weight ratio (expressed as percent). The effects of treatment (Control, HF-HED) and sex were examined using a two-way analysis of variance; as there was no effect of sex the data were reanalyzed by an unpaired Students’ *t* test between the Control and HF-HED groups. *n* = 8 Control (5 M, 3 F), *n* = 12 HF-HED (6 M, 6 F). Circles = Males, Squares = Females. *P* < 0.05 was considered significant (bolded values).

Some characteristics of the cohort, including maternal and fetal weight and fetal organ weight, have been previously described [35, 36, 53]. Maternal weight was higher in HF-HED animals on average compared to Controls 30d pre-conception and at tissue collection (*P* = 0.02 and *P* = 0.003, respectively) [53]. Maternal weight change across pregnancy was unaffected by diet. Maternal plasma glucose concentration was increased in HF-HED (*P* = 0.02) [53]. There was no difference in fetal body weight between Control and HF-HED animals (Fig. 1A); however, there was an increase in total lung weight (Fig. 1B, *P* = 0.0005) and relative lung to body weight ratio (Fig. 1C, *P* = 0.002) in the HF-HED animals compared to Controls.

### Effect of maternal HF-HED on markers of surfactant maturation, vascularization and apoptosis within the fetal lung

**Fig. 2:**
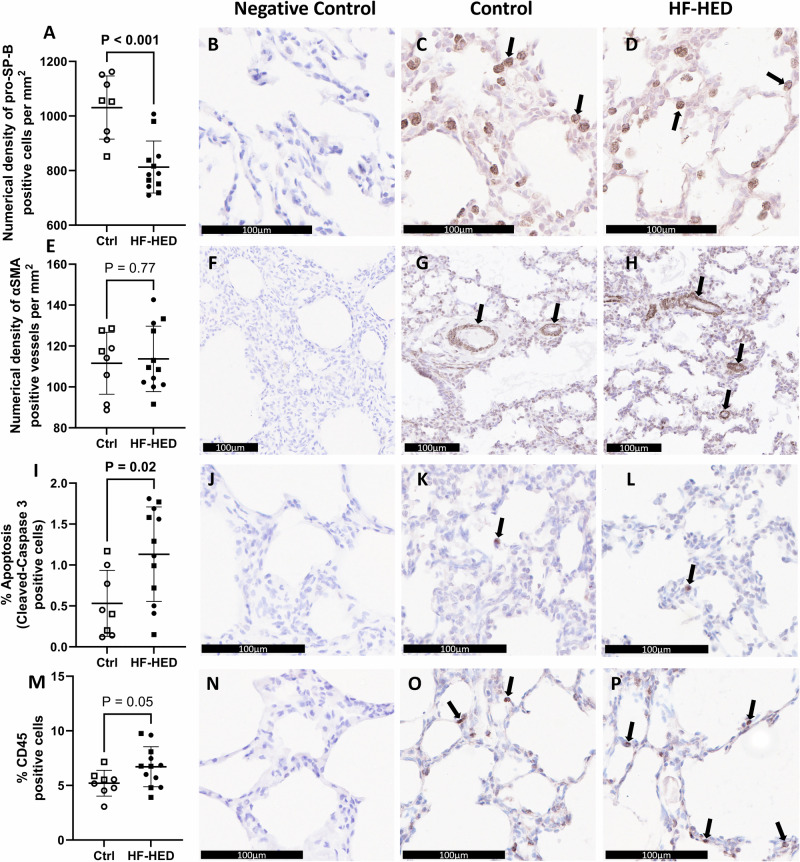
Quantification of AECII, vessel density, apoptosis and immune cells in fetal lung tissue. **A** Numerical density of pro-SP-B positive cells in the alveolar epithelium. Representative micrograph of negative control (**B**) and pro-SP-B staining from control (**C**) and HF-HED (**D**) fetal lung tissue. **E** Numerical density of αSMA stained vessels within the fetal lung. Representative micrographs of negative control (**F**) and αSMA staining from control (**G**) and HF-HED (**H**) fetal lung tissue. **I** % of apoptotic cells marked by Cleaved-Caspase 3 positive staining. Representative micrographs of negative control (**J**) and Cleaved-Caspase 3 staining from control (**K**) and HF-HED (**L**) fetal lung tissue. **M** % of immune cells marked by CD45 positive staining. Representative micrographs of negative control (**N**) and CD45 staining from control (**O**) and HF-HED (**P**) fetal lung tissue. Black arrows indicate example positive cells; Scale = 100 µm. The effects of treatment (Control, HF-HED) and sex were examined using a two-way analysis of variance; as there were no effect of sex the data were reanalyzed by an unpaired Students’ *t* test between the Control and HF-HED groups. *n* = 8 Control (5 M, 3 F), *n* = 12 HF-HED (6 M, 6 F). Circles = Males, Squares = Females. *P* < 0.05 was considered significant (bolded values).

**Fig. 3:**
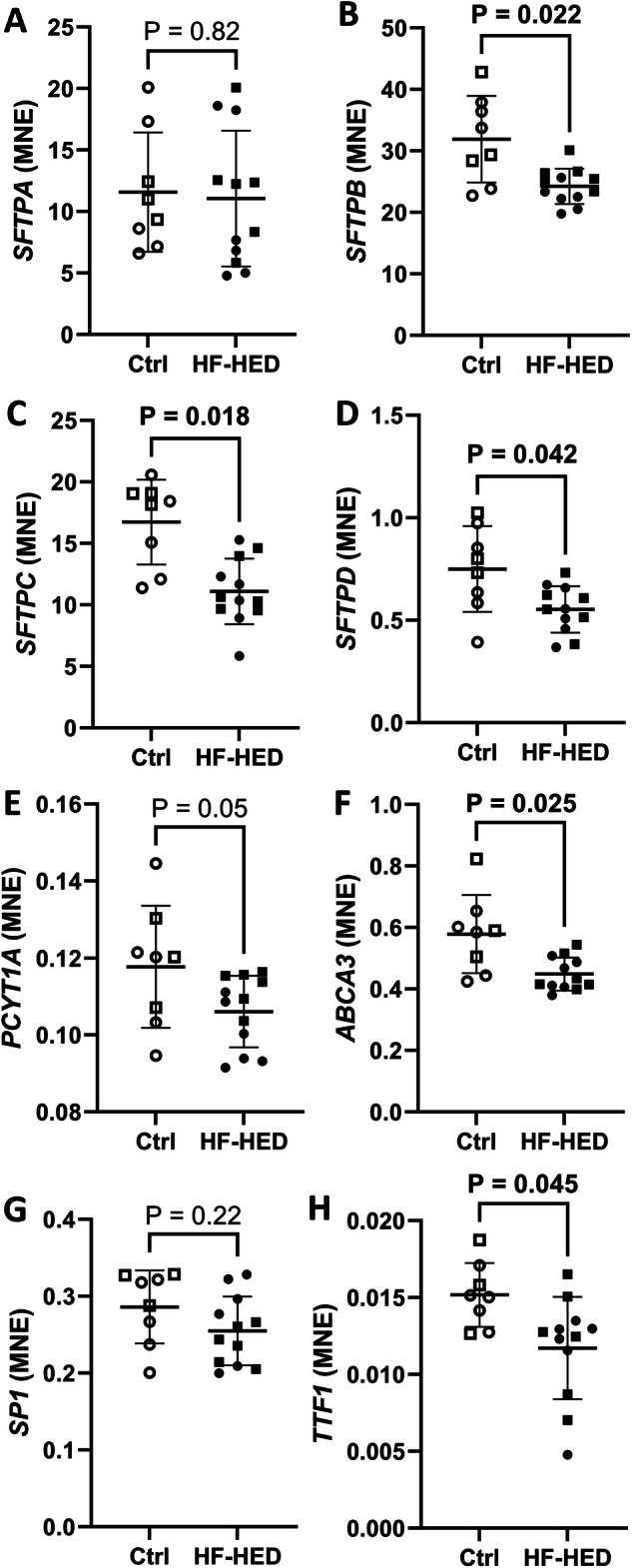
Surfactant maturation markers mRNA expression in control and HF-HED fetal lungs. **A** *SFTPA*, **B** *SFTPB*, **C** *SFTPC*, **D** *SFTPD*, **E** *PCYT1A*, **F** *ABCA3*, **G** *SP1*, **H** *TTF1*. The effects of treatment (Control, HF-HED) and sex were examined using a two-way analysis of variance; as there was no effect of sex the data were reanalyzed by an unpaired Students’ *t* test between the Control and HF-HED groups. *n* = 8 Control (5 M, 3 F), *n* = 12 HF-HED (6 M, 6 F). Circles = Males, Squares = Females. *P* < 0.05 was considered significant (bolded values). MNE mean normalized expression.

There was a decrease in the number of type II AECs within the alveolar epithelium of the lungs of fetal baboons exposed to maternal HF-HED (Fig. 2A, *P* < 0.001). However, there was no difference in the density of αSMA positive vessels in the HF-HED group compared to Controls (Fig. 2E). In line with the decreased number of type II AECs, mRNA expression of *SFTPB* (Fig. 3B, *P* = 0.022), *SFTPC* (Fig. 3C, *P* = 0.017), *SFTPD* (Fig. 3D, *P* = 0.042), *PCYT1A* (Fig. 3E, *P* = 0.05), *ABCA3* (Fig. 3F, *P* = 0.025) and *TTF1* (Fig. 3H, *P* = 0.044) were all decreased in the lungs of HF-HED exposed fetuses compared to Controls. There was no difference in *SFTPA* (Fig. 3A) or *SP1* (Fig. 3G) mRNA expression between Control and HF-HED group.

### Effect of maternal HF-HED on markers of water and sodium movement in the fetal lung

**Fig. 4:**
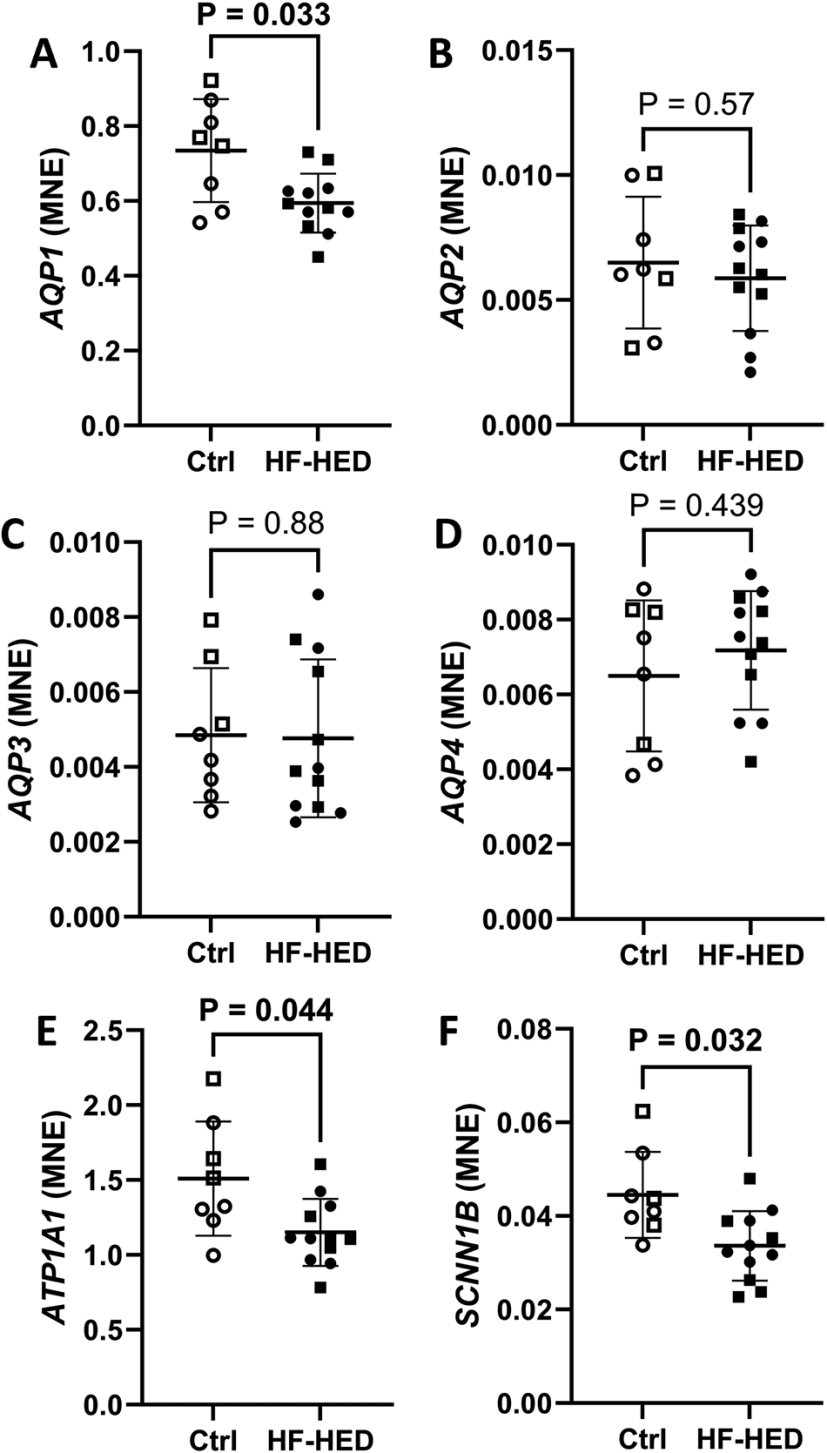
Water and sodium movement markers mRNA expression in control and HF-HED fetal lungs. **A** *AQP1*, **B** *AQP2*, **C** *AQP3*, **D** *AQP4*, **E** *ATP1A1*, **F** *SCNN1B*. The effects of treatment (Control, HF-HED) and sex were examined using a two-way analysis of variance; as there was no effect of sex the data were reanalyzed by an unpaired Students’ *t* test between the Control and HF-HED groups. *n* = 8 Control (5 M, 3 F), *n* = 12 HF-HED (6 M, 6 F). *P* < 0.05 was considered significant (bolded values). Circles = Males, Squares = Females. MNE mean normalized expression.

The mRNA expression of *AQP1* (Fig. 4A, *P* = 0.033), *ATP1A1* (Fig. 4E, *P* = 0.044) and *SCNN1B* (Fig. 4F, *P* = 0.032) were decreased in fetal lungs of maternal HF-HED compared to Controls. The mRNA expression of *AQP2* (Fig. 4B), *AQP3* (Fig. 4C), and *AQP4* (Fig. 4D) mRNA expression was unchanged in HF-HED compared to Controls.

### Effect of maternal HF-HED on upstream regulators of fetal lung maturation

**Fig. 5:**
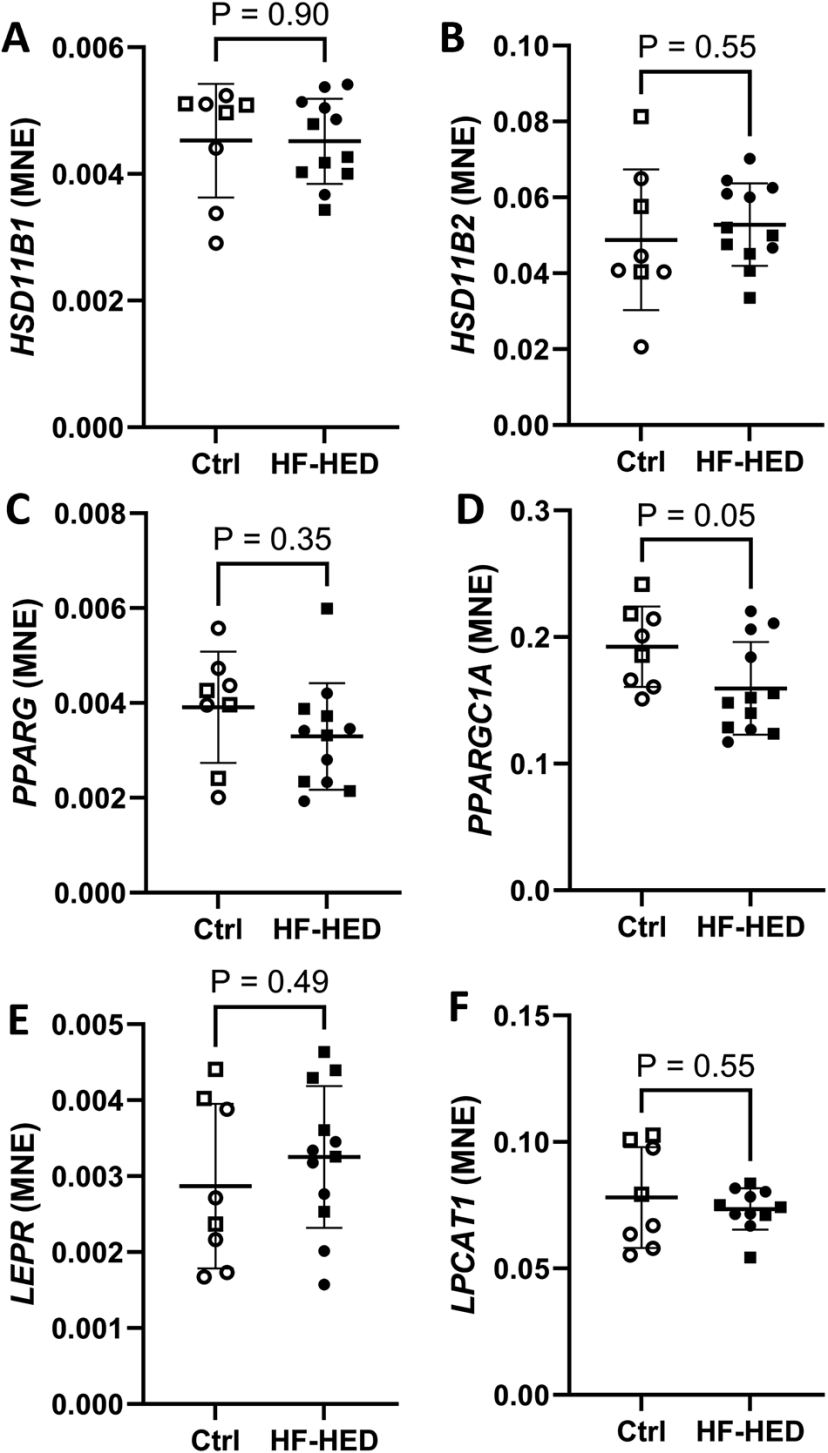
Glucocorticoid conversion and PPARγ signaling mRNA expression in control and HF-HED fetal lungs. **A** *HSD11B1*, **B** *HSD11B2*, **C** *PPARG*, **D** *PPARGC1A*, **E** *LEPR*, **F** *LPCAT1*. The effects of treatment (Control, HF-HED) and sex were examined using a two-way analysis of variance; as there was no effect of sex the data were reanalyzed by an unpaired Students’ *t* test between the Control and HF-HED groups. *n* = 8 Control (5 M, 3 F), *n* = 12 HF-HED (6 M, 6 F). Circles = Males, Squares = Females. *P* < 0.05 was considered significant (bolded values). MNE mean normalized expression.

There was no change in the mRNA expression of enzymes involved in cortisol and cortisone conversion; *HSD11B1* (Fig. 5A)*, HSD11B2* (Fig. 5B), or PPARγ signaling; LEPR (Fig. 5C), PPARG (Fig. 5D), PPARGC1A (Fig. 5E), in the fetal lung in response to maternal HF-HED.

### Effect of maternal HF-HED on glucose and fatty acid synthesis and transport within the fetal lung

**Fig. 6:**
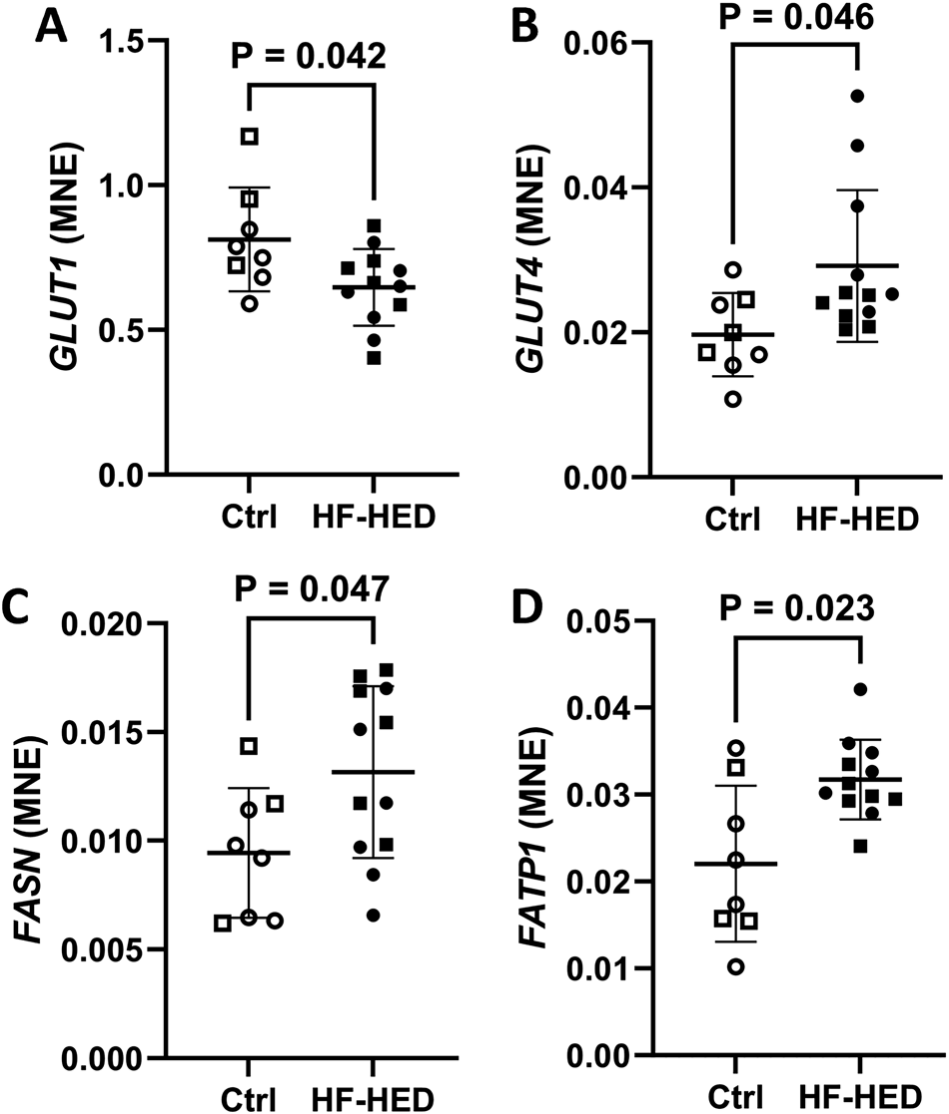
Glucose and fatty acid transport and synthesis mRNA expression in control and HF-HED fetal lungs. **A** GLUT1, **B** GLUT4, **C** FASN, **D** FATP1. The effects of treatment (Control, HF-HED) and sex were examined using a two-way analysis of variance; as there was no effect of sex the data were reanalyzed by an unpaired Students’ *t* test between the Control and HF-HED groups. *n* = 8 Control (5 M, 3 F), *n* = 12 HF-HED (6 M, 6 F). Circles = Males, Squares = Females. *P* < 0.05 was considered significant (bolded values). MNE mean normalized expression.

The mRNA expression of *GLUT1* (Fig. 6A, *P* = 0.042) was downregulated, whereas *GLUT4* (Fig. 6B, *P* = 0.046) was upregulated in the fetal lung in maternal HF-HED compared to Controls. mRNA expression of FASN and FATP1 (Fig. 6C, D, *P* = 0.047, *P* = 0.023, respectively) was both upregulated in the fetal lung in response to maternal HF-HED.

### Effect of maternal HF-HED on inflammation and immune cell markers within the fetal lung

**Fig. 7:**
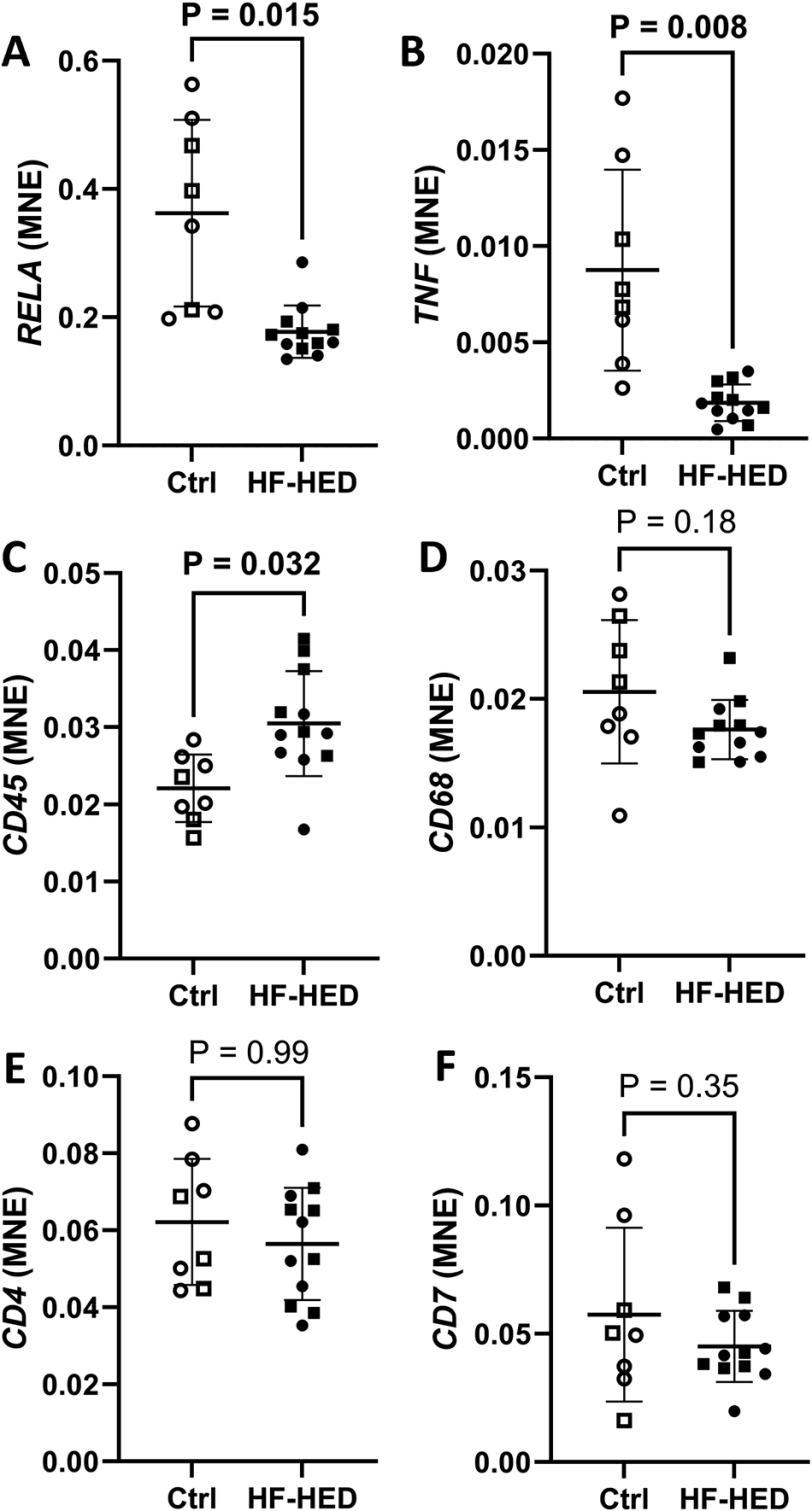
Immune marker mRNA expression in control and HF-HED fetal lungs. **A** *RELA*, **B** *TNF*, **C** *CD45*, **D** *CD68*, **E** *CD4*, **F** *CD7*. The effects of treatment (Control, HF-HED) and sex were examined using a two-way analysis of variance; as there was no effect of sex the data were reanalyzed by an unpaired Students’ *t* test between the control and HF-HED groups. *n* = 8 Control (5 M, 3 F), *n* = 12 HF-HED (6 M, 6 F). Circles = Males, Squares = Females. *P* < 0.05 was considered significant (bolded values). MNE mean normalized expression.

mRNA expression of inflammatory markers, *RELA* and *TNF*, was reduced in the fetal lungs of HF-HED animals compared to Controls (Fig. 7A, B, *P* = 0.015, *P* = 0.008). mRNA expression of general immune cell marker *CD45* (Fig. 7C, *P* = 0.031) was upregulated in HF-HED lungs compared to Controls. However, there was no difference in the density of CD45-positive immune cells present in fixed tissue samples (Fig. 2M, *P* = 0.05). There was no change in the expression of B and T cell markers *CD68, CD4*, and *CD7* in response to maternal HF-HED.

## Discussion

This is the first study in the clinically relevant non-human primate that demonstrates impairment of fetal lung development in response to maternal high-fat high-energy diet. Consistent with our previous studies in the sheep fetus during late gestation [15]. We observed a reduction in AECII within the alveolar epithelium (Fig. 2A), reducing the capacity for pulmonary surfactant production. This reduction in AECII was also associated with reduced surfactant protein gene expression (*SFTP-B, SFTP-C, SFTP-D*, Fig. 3B–D) in addition to rate-limiting enzymes in the de novo production of surfactant phospholipids (*PCYT1A* and *ABCA3*, Fig. 3E, F). Phosphate cytidylyltransferase 1, Choline, Alpha (*PCYT1A*), also known as choline-phosphate cytidylyltransferase alpha (CCTα) in humans, is a key rate-limiting enzyme in the production of phosphatidylcholine and dipalmitoylphosphatidylcholine, the most abundant surfactant phospholipids necessary for reducing the surface tension of the surfactant film and preventing alveolar collapse [54]. These results indicate that fetal lungs exposed to a HF-HED not only have a reduced capacity to produce surfactant via a lower number of surfactant-producing cells, but may also have a reduced capacity to lower the surface tension at the alveolar air-liquid interface at birth due to decreased synthesis of phospholipids and impaired phospholipid insertion, thereby increasing the risk of developing neonatal respiratory complications. In addition to surfactant maturation, the reabsorption of lung liquid at birth is crucial to the transition to air breathing and is reliant on key ion and water channels to facilitate this process. To our knowledge we are the first to discover a decrease in aquaporin (Fig. 4A) and sodium transporter mRNA expression (Fig. 4E, F) in the fetal lung in response to maternal obesogenic diet. Impaired reabsorption of lung liquid can result in TTN and the development of RDS. TTN is also related to preterm delivery, which is observed at a higher frequency in women that are living with obesity during pregnancy, experiencing maternal diabetes, or due to changes in steroid metabolism in the maternal-placental-fetal unit [6]. Indeed, a retrospective cohort study found that infants of mothers with obesity (*n* = 7200) had a higher incidence of TTN (14.3% vs. 11.6%; *P* < 0.001) than infants of normal BMI mothers (*n* = 14,998), and subsequently developed RDS (7.2% vs. 6.1%; *P* = 0.002) at a higher rate than infants of normal BMI mothers [7]. The combined increased risk of developing TTN and RDS may explain the larger proportion of sudden unexpected infant death observed in pregnancies with obesity [55].

The lack of change in mRNA expression of *HSD11B-1* and *HSD11B-2* (Fig. 5A, B) indicates that HF-HED had negligible effect on glucocorticoid availability or action in the lung of fetuses. In the normal developing lung, cortisol plays the most crucial role in preventing RDS by increasing surfactant phospholipid production and thinning the alveolar walls [56]. However, it has been proposed that insulin antagonizes the stimulatory effects of glucocorticoids on pulmonary surfactant metabolism in a dose-dependent manner [57]. In addition, we have previously reported decreased expression of genes regulating glucocorticoid availability (*HSD11B-1* and *NR3C1)* in the fetal sheep lung after a 10-day glucose infusion [17]. Hence, it may be that if plasma insulin concentration was increased by greater severity or duration during the HF-HED protocol, as seen with GDM in pregnancies with overweight and obesity in humans, a decrease in cortisol availability in the lung may be observed. Lung alveolarization is also driven by angiogenesis and the formation of a capillary network to provide the platform for gas exchange [58]. Interruption of this temporally sensitive process can result in permanent deficits in lung development and function. Previous evidence from rodent models of high-fat diet during pregnancy has demonstrated reduced angiogenesis in offspring lungs [59]. This response was absent in our study, measured by the density of αSMA-positive vessels in the alveolar epithelium. One possible explanation involves adipokines such as Leptin, which was not measured in this study. Leptin affects angiogenesis via signaling through the cognate receptor Ob-R. Although acute increases in plasma leptin levels can be proangiogenic, chronic elevation of leptin concentrations, such as those found in instances of people living with obesity, is associated with attenuated angiogenic responses through upregulation of protein tyrosine phosphatase B1 (PTP1B) [60]. However, in this study, there was no change in leptin-responsive genes and the PPARγ signaling pathway (*LEPR*, *PPARG, PPARGC1;* Fig. 5C–E), indicating no change in leptin signaling within the fetal lungs.

The increased expression of key genes involved in fatty acid metabolism, fatty acid synthase (*FASN*) and fatty acid transport protein (*FATP1*; Fig. 6C, D), in HF-HED fetal lungs suggests a potential increase in the capacity for uptake of fatty acids via transporters and the synthesis of fatty acids within the lung. In particular, increased expression of *FASN* may have a direct impact upon the production of surfactant phospholipids, since fatty acid synthase is a key enzyme in the production of palmitate, a major component utilized in surfactant phospholipid production [61]. The induction of *GLUT1* in the lungs is glucose-mediated and is usually upregulated by decreasing plasma glucose concentrations [62]. In contrast, *GLUT4* is mediated by changes in plasma insulin concentration [63]. The decrease in *GLUT1* and increase in *GLUT4* mRNA expression in response to HF-HED (Fig. 6A, B) is therefore explained by the increased maternal plasma glucose and insulin concentration. Given the negative impact insulin has on the effects of glucocorticoids in the production of pulmonary surfactant [57]. this change in glucose transporter expression may be representative of the observed negative impact on surfactant maturation.

Interestingly, there was an increase in apoptosis detected in the lungs of HF-HED animals compared to Controls (Fig. 2I). This was in agreement with previous studies of maternal high-fat diet in rats, where apoptosis was measured by TUNEL and cleaved caspase expression [26]. Increased lung apoptosis is observed in cases of acute lung injury and acute RDS and is often associated with the production of pro-inflammatory cytokines such as TNFα. However, we saw the opposite expression profile with a reduction in both *RELA* (NFκB signaling) and *TNF* mRNA expression. We did, however, observe an increase in immune cells present in the lungs measured by CD45 staining (Fig. 2M) and mRNA expression (Fig. 7C). There are a number of possible explanations for this mismatch in data, such as negative feedback of the prolonged inflammatory state and the concept of the “overcompensation response” where some pregnancies with obesity have a lowered placental cytokine signaling and overall inflammatory signature [64, 65]. Given that there was no difference in specific immune cell markers CD4 (memory and regulatory T-cell co-receptor), CD7 (T-cell and NK cells) and CD68 (monocyte lineage), coupled with the decrease in pro-inflammatory markers, the observed increase in CD45 may represent amplified resident pulmonary immune cells such as macrophages and dendritic cells. The inflammatory response in individuals with obesity is incredibly varied, with tissue specific responses, in addition to differences in the composition of the diet used to induce and obesogenic phenotype, the timing, the duration for which the diet is fed.

There are limitations to this study. As this study was not originally designed with lung development as the main outcome, we were unable to assess some key markers of surfactant maturation of offspring; for example, bronchoalveolar lavage fluid was not collected and therefore surfactant phospholipid analysis could not be performed. Though the study was not sufficiently powered to look at the effect of sex, we did initially analyze all data using a two-way ANOVA with this outcome in mind. While some interesting trends were observed, none of the data reached statistical significance and thus sexes were pooled within treatment groups. Further studies examining the effect of offspring sex are required, given the large evidence base for sexually dimorphic outcomes within other organs in response to HF-HED [35, 53] as well as our model of nutrient restriction during pregnancy [29, 66, 67]. Given the limited tissue available, we chose to extract RNA rather than protein, as this allowed us to examine a greater range of molecular targets. In addition, we have previously demonstrated a tight correlation between the expression of markers of surfactant maturation between qRT-PCR and Western blot techniques [23, 68]. Lastly, lung tissue was not inflation fixed, therefore we could not assess the airspace ratio.

## Conclusion

Taken together, these data overwhelmingly indicate that a maternal high-fat high-energy diet during late gestation can result in increased risk of respiratory complications at birth and may be an additional risk factor for premature infants that already have a high risk of RDS. Surfactant maturation was negatively impacted, with a reduction in the number of type II alveolar epithelial cells, reduced expression in rate-limiting enzymes in the production of surfactant phospholipids (*PCYT1A, ABCA3*), and reduced expression of surfactant proteins (*SFTPB, SFTPC, SFTPD*). Lung liquid reabsorption was also negatively impacted with reduced expression of sodium transporters (*ATP1A1* and *SCNN1*) and may be associated with the higher incidence of TTN in pregnancies with obesity. Glucose and fatty acid metabolism were dysregulated in the fetal lung with a downregulation of glucose transporter 1 (*GLUT1*) but an upregulation of insulin-dependent glucose and fatty acid transporters (*GLUT4, FATP1*) and fatty acid synthesis (*FASN*). The impact on the fetus in response to maternal obesogenic diet involves a combination of factors related to the hyperglycemic and proinflammatory environment. The impact of these factors independently on fetal and neonatal lung development is therefore difficult to tease apart, programming distinct effects at both the structural and molecular levels. It is therefore crucial that further detailed analysis of these individual factors is performed to understand how they work synergistically or antagonistically to regulate the respiratory outcomes of these neonates at birth and in postnatal life. Identifying risk factors associated with maternal obesogenic diet and the underlying mechanisms will improve our ability to predict and treat negative outcomes.

## Data Availability

The data generated and analyzed during this study are available from the corresponding author on reasonable request.
